# *QuickStats*: Percentage* of Currently Employed Adults Aged ≥18 Years Who Had Paid Sick Leave Benefits^†^ at Last Week’s Job or Business, by Region — National Health Interview Survey, United States, 2019 and 2020^§^

**DOI:** 10.15585/mmwr.mm7117a4

**Published:** 2022-04-29

**Figure:**
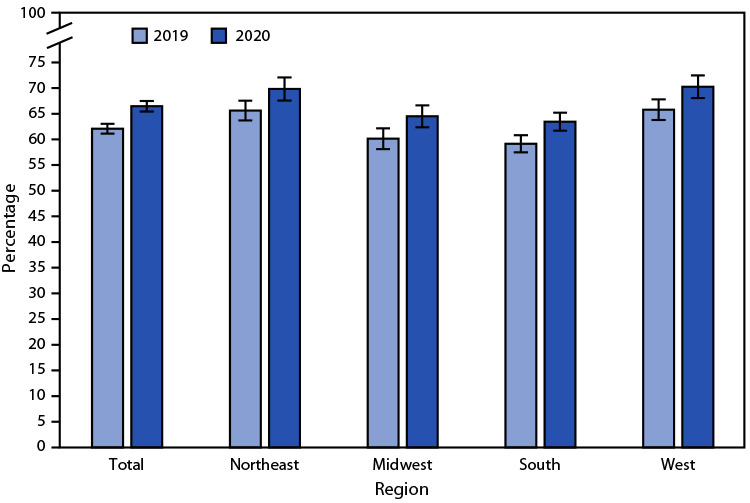
The percentage of currently employed adults who had access to paid sick leave increased from 62.1% in 2019 to 66.5% in 2020. During this period, increases were noted among all regions of the country (Northeast: from 65.6% to 69.8%, Midwest: from 60.1% to 64.5%, South: from 59.1% to 63.5%, and West: from 65.8% to 70.3%). In both years, rates were highest in the Northeast and West and lowest in the Midwest and South.

**Source:** National Health Interview Survey, 2019–2020 data. https://www.cdc.gov/nchs/nhis.htm

For more information about this topic, CDC recommends the following link: https://blogs.cdc.gov/niosh-science-blog/2012/07/30/sick-leave/

